# Comparison of the effects of extracorporeal shock wave therapy and a vacuum erectile device on penile erectile dysfunction: a randomized clinical trial: Retraction

**DOI:** 10.1097/MD.0000000000010262

**Published:** 2018-03-23

The article, “Comparison of the effects of extracorporeal shock wave therapy and a vacuum erectile device on penile erectile dysfunction: a randomized clinical trial”^[1]^, which appeared in Volume 96, Issue 44 of *Medicine*, is being retracted for plagiarized data previously published in another study.
